# Author Correction: Enzyme mediated synthesis of hybrid polyedric gold nanoparticles

**DOI:** 10.1038/s41598-022-15652-2

**Published:** 2022-07-05

**Authors:** Célia Arib, Jolanda Spadavecchia, Marc Lamy de la Chapelle

**Affiliations:** 1 CNRS, UMR 7244, CSPBAT, Laboratoire de Chimie, Structures et Propriétés de Biomatériaux Et D’Agents Thérapeutiques Université Paris 13, Sorbonne Paris Cité, Bobigny, France; 2 Institut Des Molécules et Matériaux du Mans (IMMM-UMR CNRS 6283), Le Mans Université, Avenue Olivier Messiaen, 72085 Le Mans Cedex 9, France

Correction to: *Scientific Reports* https://doi.org/10.1038/s41598-021-81751-1, published online 05 February 2021

The original version of this Article contained errors in Figure 3 where the histograms were incorrect in panels (a1) and (b1). The original Figure 3 and accompanying legend appear below.

**Figure 3:**
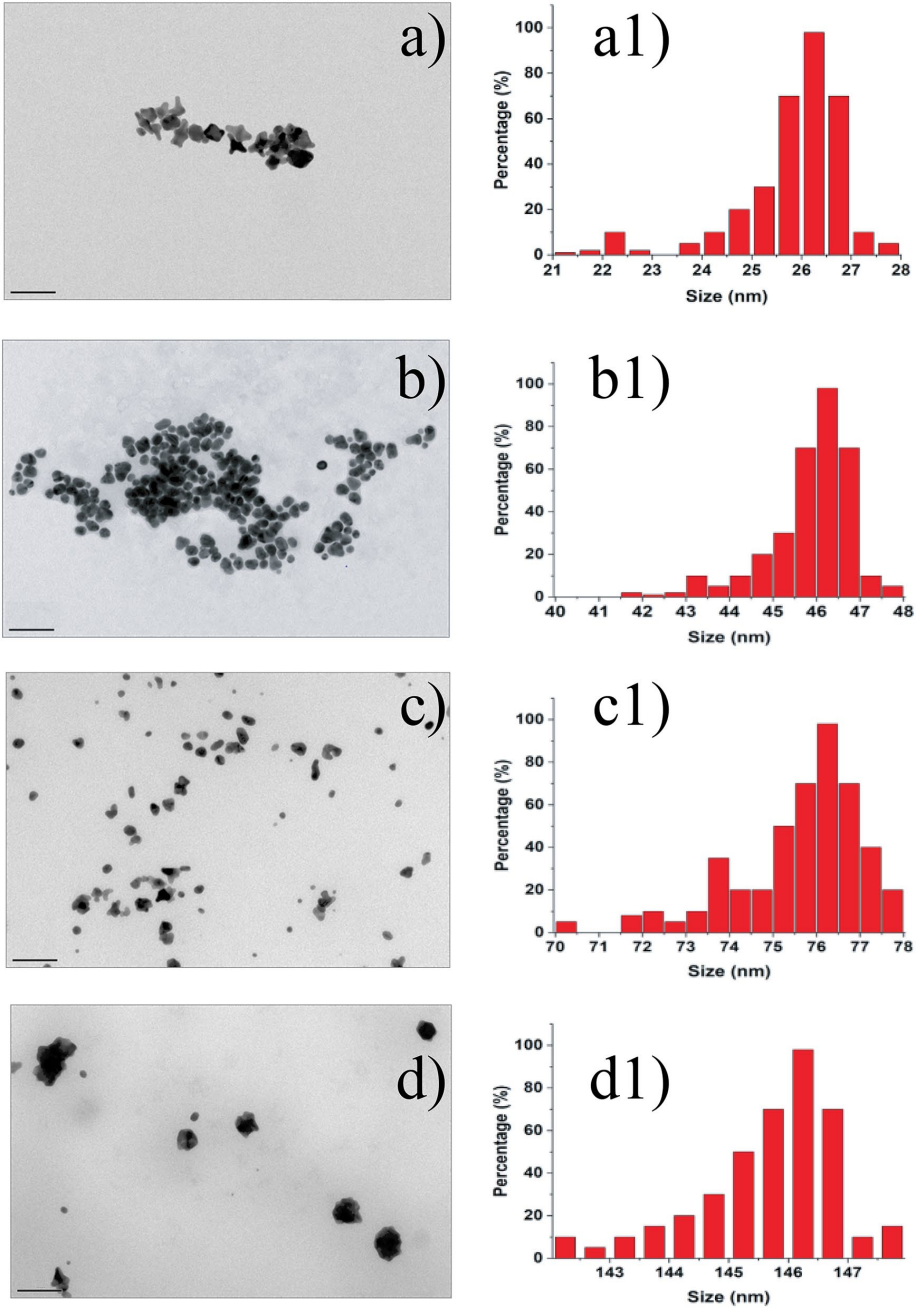
Nanoparticle morphology. TEM images and size distribution of gold nanoparticles produced with HEPES (**a**–**a1**), MnSOD (**b**–**b1**), CAT (**c**–**c1**) and BSA (**d**–**d1**). The scale bar corresponds to 50 nm on each image.

The original Article has been corrected.

